# Supplementary material for: Chronic inflammation degrades CD4 T cell immunity to prior vaccines in treated HIV infection
Source: Nat Commun. 2024 Nov 25;15:10200. doi: 10.1038/s41467-024-54605-3 (PMC11589758; doi:10.1038/s41467-024-54605-3)
Supplement: Supplementary file 1 — Supplementary Information [file 41467_2024_54605_MOESM1_ESM.pdf]

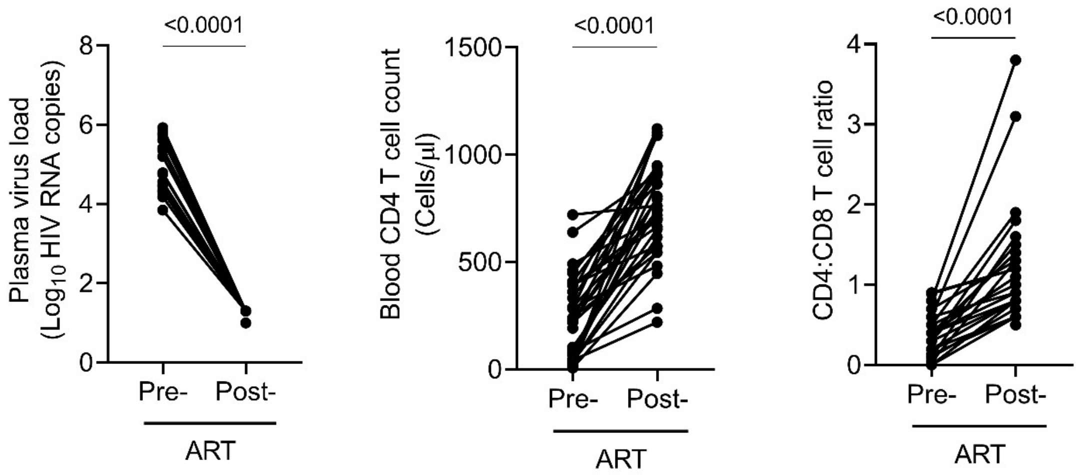

Supplementary Figure 1: Virus load, CD4 T cell count and CD4:CD8 T cell ratio before and after cART. Pre-cART refers to values at start of cART and post-cART refers to values at recruitment in this study. Circles represent individual participants (32 HIV). Median and IQR are presented, statistical analysis between groups was performed by Mann-Whitney test and P values are shown. Source data are provided as a Source Data file.

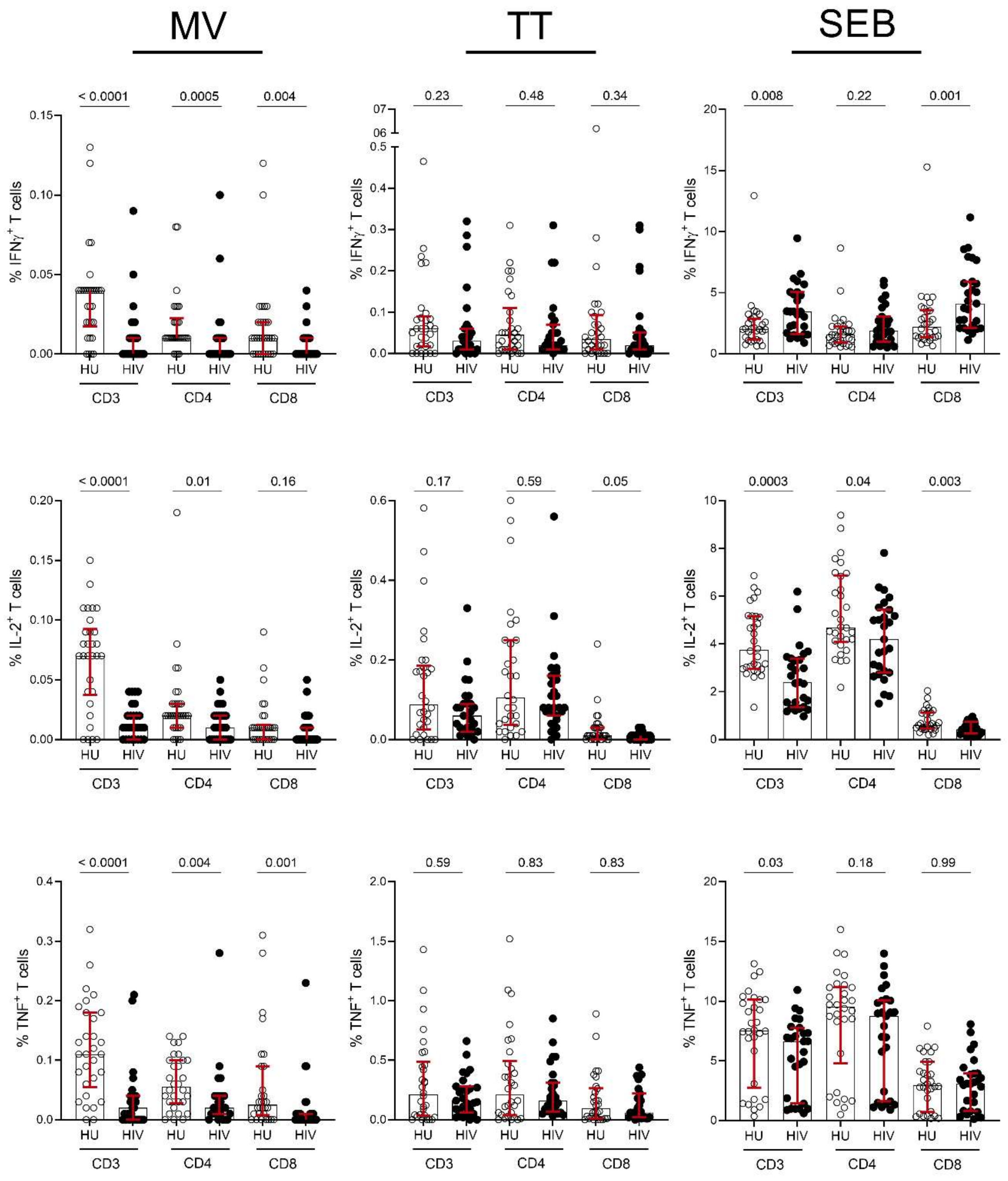

Supplementary Figure 2: Breakdown of individual cytokine responses to TT, MV and SEB stimulations, related to Figure 1. Circles represent individual participants of the uninfected (HU; 30) and HIV-infected (HIV; 27) groups. Median and IQR are presented, statistical analysis between groups was performed by Mann-Whitney test and P values are shown. Source data are provided as a Source Data file.

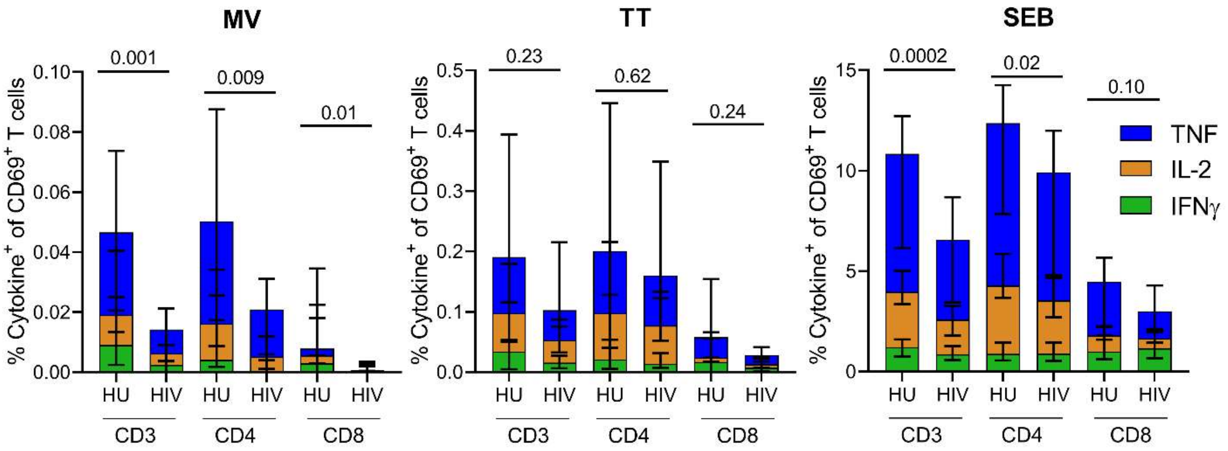

Supplementary Figure 3: Percentages of IFNγ⁺, IL-2⁺ and TNF⁺ cells of CD3 CD69⁺, CD4 CD69⁺ or CD8 CD69⁺ T fractions after stimulation with MV, TT or SEB, **related to Figure 1.** Circles represent individual participants of the uninfected (HU; 30) and HIV-infected (HIV; 27) groups. Median and IQR are presented, statistical analysis between groups was performed by Mann-Whitney test and P values are shown. Source data are provided as a Source Data file.

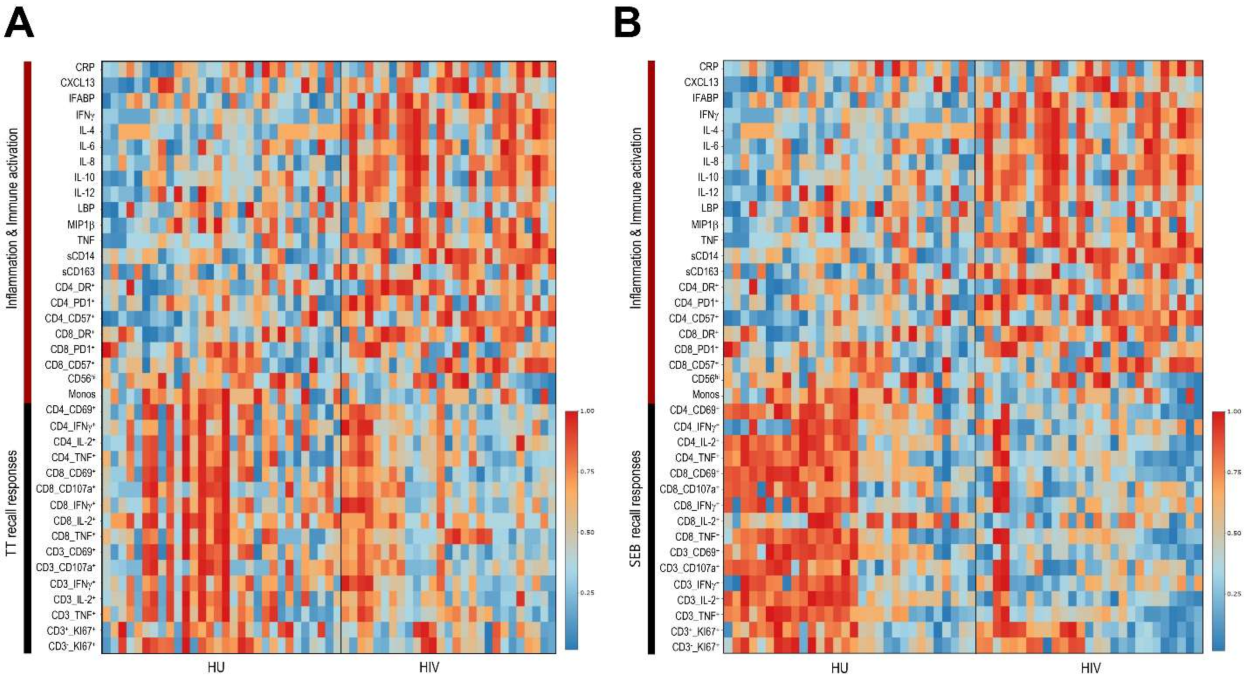

Supplementary Figure 4: Heatmap integrating all inflammation and immune activation measures with *in vitro* T cell responses to TT (A) or SEB (B) stimulation, related to Figure 3. Percentized expression levels across donors are presented for each variable and colors indicate the highest (red) or lowest (blue) expression level for a given marker.

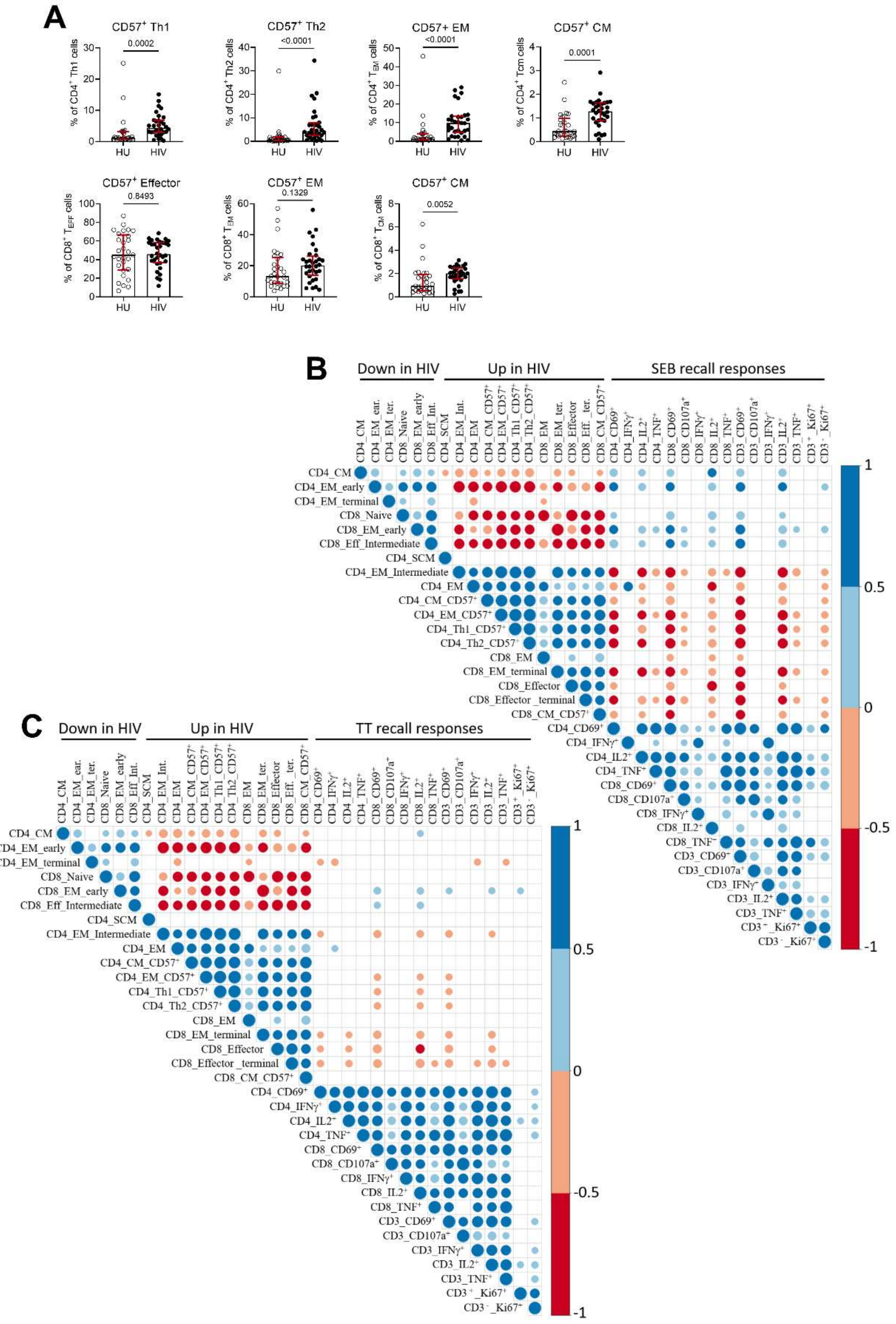

Supplementary Figure 5: (A) CD57 expression on T cell subsets. Circles represent individual participants of the uninfected (HU; 29) and HIV-infected (HIV; 32) groups. **(B-C) Correlogram of T cell responses after TT and SEB stimulations in relation to T cell subsets frequencies, related to Figure 4.** Colored circles represent correlations with $P \leq 0.05$ as determined by Spearman analysis. Blue and red circles indicate positive and negative correlations respectively. Color intensity and the size of the circle are proportional to the correlation coefficients. The corresponding colors of the correlation coefficients are presented in the legend. Source data are provided as a Source Data file.

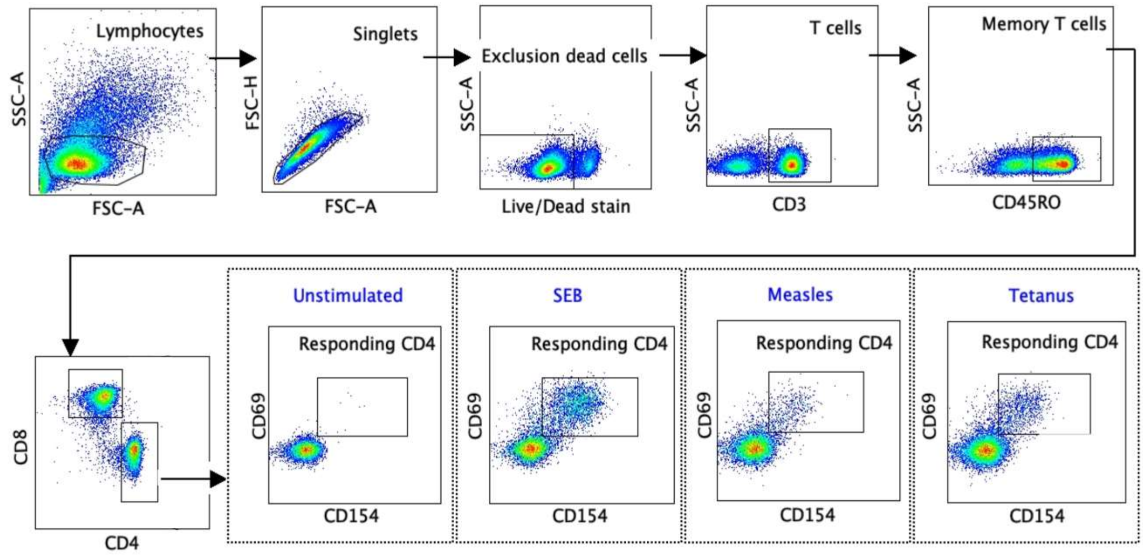

Supplementary Figure 6: Gating strategy of sorted CD4 T cells after MV or TT stimulation. After gating of memory CD4 T cells ($CD3^+$ $CD45RO^+$ $CD4^+$), responding cells were identified as $CD154^+$ and $CD69^+$ whereas non-responsive cells were $CD154^-$ and $CD69^-$. Unstimulated and SEB conditions were used to set gates followed by cell sorting of $CD154^+CD69^+$ or $CD154^-CD69^-$ for the unstimulated, MV and TT conditions.

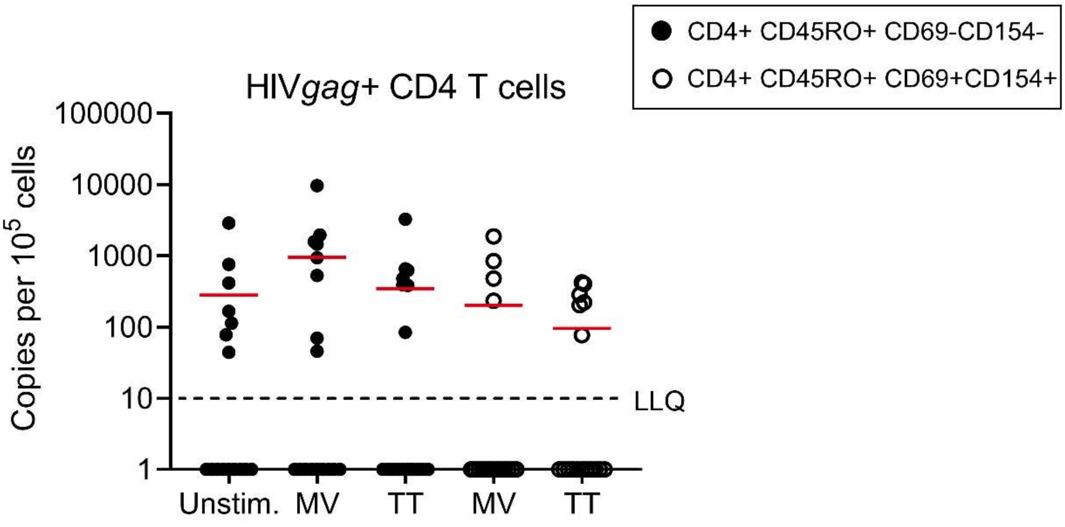

n *gag*+	7/16	8/17	7/17	4/17	6/17
% *gag*+	43.8	47.1	41.2	23.5	35.3

Supplementary Figure 7: Copies of HIV gag DNA in CD154^{+} and CD154^{-} CD4 T cells sorted after PBMC stimulation with MV or TT. Circles represent up to 17 HIV participants and both the number and percentages of HIV *gag* positive are shown under the figure. The mean copies per 10^5 cells is shown and a dotted line indicates the LLQ (lower limit of quantification). Source data are provided as a Source Data file.

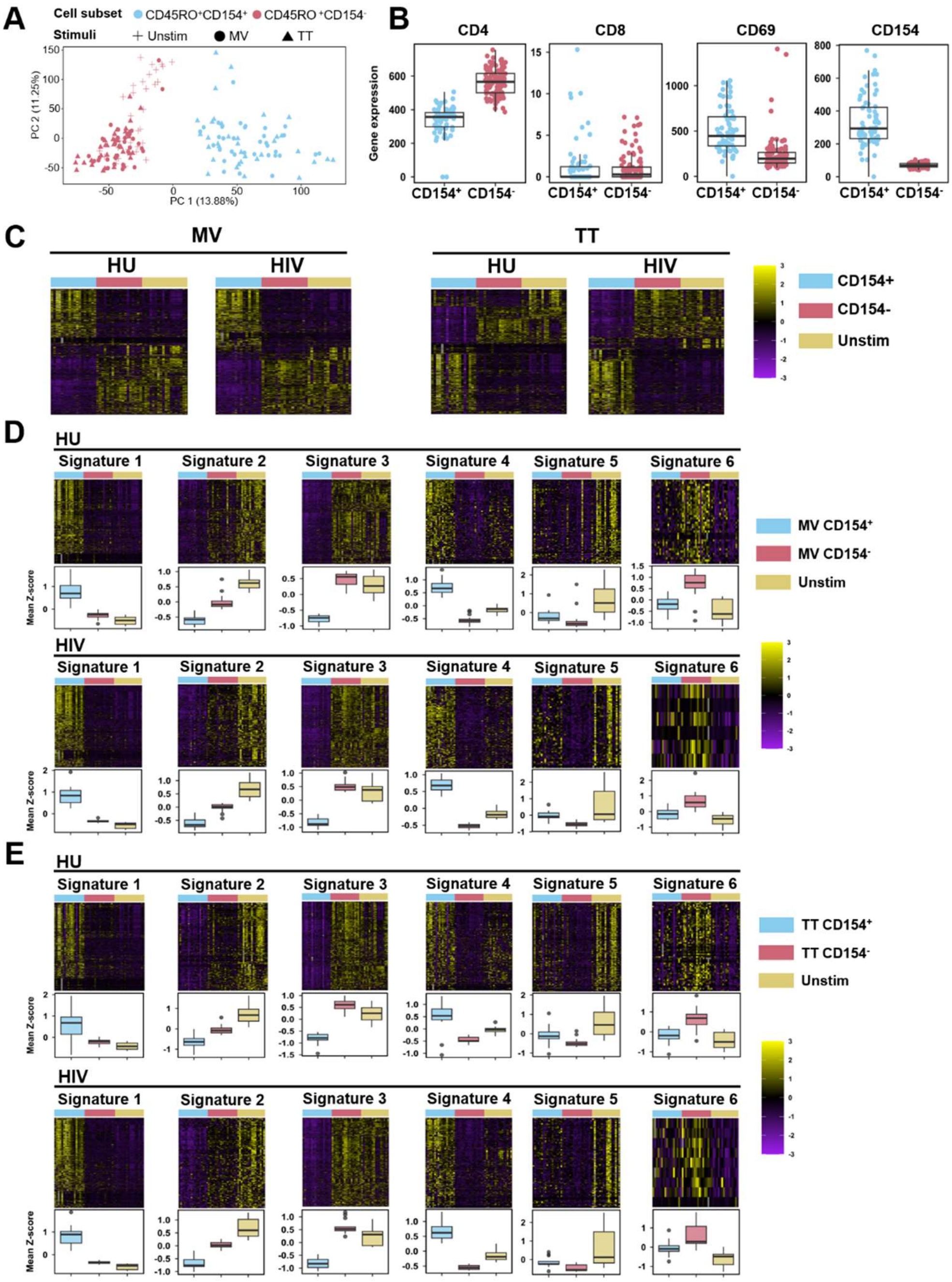

Supplementary Figure 8: Transcriptome profiles of CD154^{+} and CD154^{-} CD4 T cells sorted after PBMC stimulation with MV or TT. (**A**) Principal component analysis of normalized gene counts of CD154^{+} or CD154^{-} memory CD4 T cells sorted after MV or TT stimulation. (**B**) Expression of CD4, CD8, CD154 and CD69 in the sorted CD4 T cell subsets. (**C**) Heatmaps and box plots of gene signature profiles of uninfected and HIV-infected groups after MV stimulation. Blue and red colors indicate CD154^{+} or CD154^{-} memory CD4 T cells sorted after MV stimulation. Yellow color indicate unstimulated condition. Yellow and purple shades in the heatmap indicates up and downregulated genes respectively. (**D**) Heatmaps of genes with significant differential expression after MV or TT stimulation compared to unstimulated condition in the HIV-infected or uninfected groups. (**E**) Heatmaps and box plots of gene signature profiles of HIV-infected or uninfected groups after TT stimulation. Blue and red colors indicate CD154^{+} or CD154^{-} memory CD4 T cells sorted after TT stimulation. Yellow color indicate unstimulated condition. Yellow and purple shades in the heatmap indicates up and downregulated genes respectively.

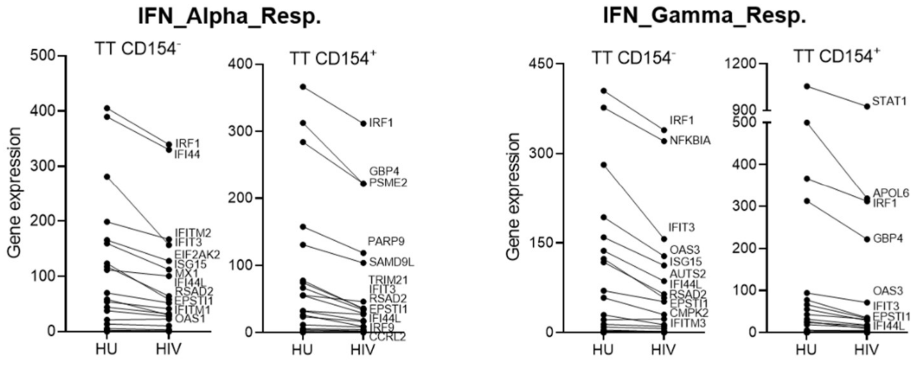

Supplementary Figure 9, related to Figure 6: Median expression of the top 20 most downregulated genes by Log_2_Fold of the HALLMARK_IFN_ALPHA_RESPONSE and HALLMARK_IFN_GAMMA_RESPONSE pathways in TT stimulated CD4 T cells. Source data are provided as a Source Data file.

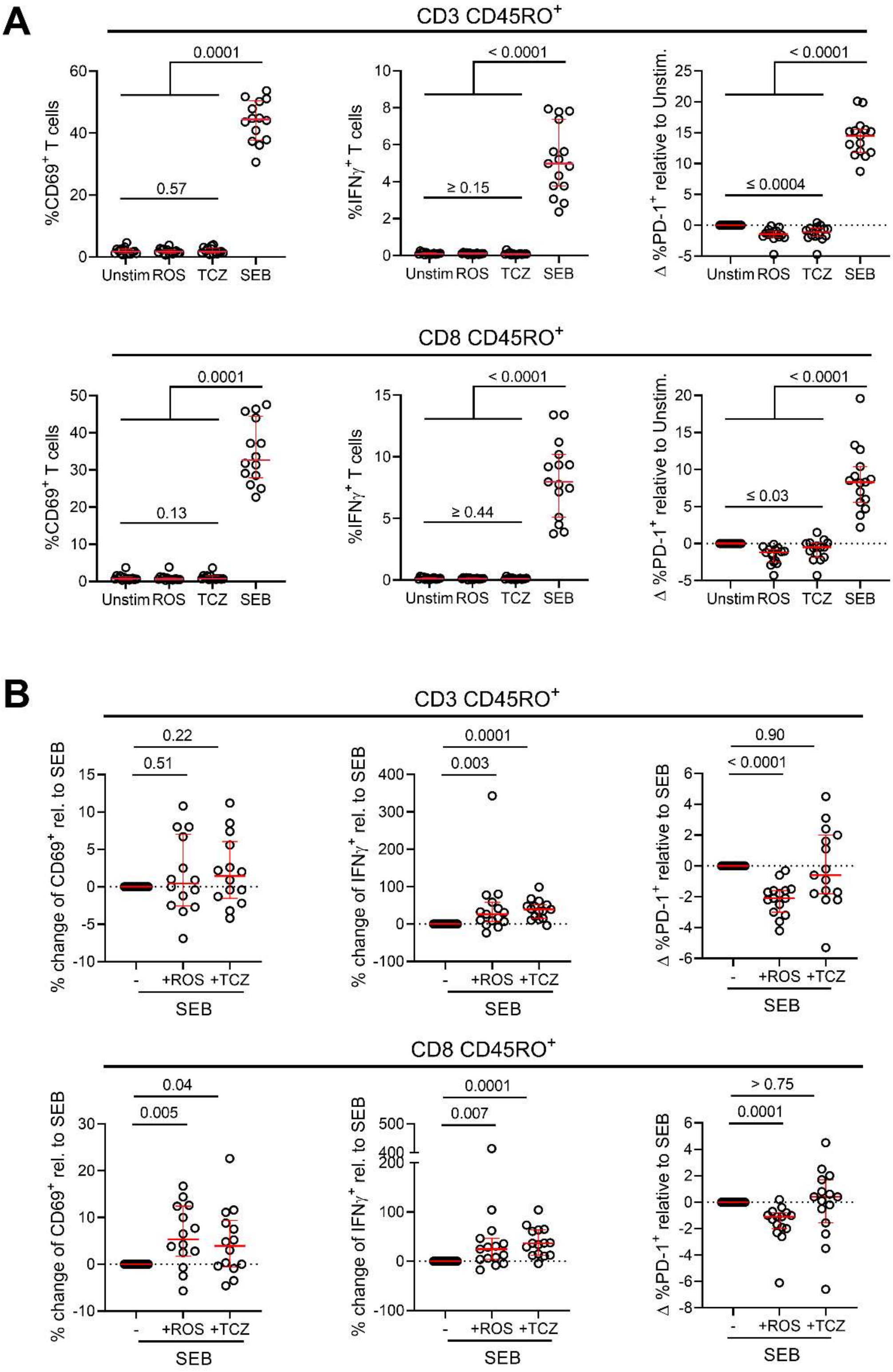

Supplementary Figure 10, related to Figure 7: (A) Percentages of CD69+, IFNγ+ and difference in percentages of PD1+ memory CD3 and CD8 T cells upon stimulation with rosuvastatin (ROS), tocilizumab (TCZ) or SEB compared to unstimulated condition. (**B**) Difference in percentages of CD69+, IFNγ+ and PD1+ memory CD3 and CD8 T cells upon stimulation with SEB+ROS or SEB+TCZ compared to SEB alone. Comparison between unstimulated, SEB, ROS or TCZ conditions in 15 ART-treated people living with HIV was performed by Wilcoxon paired test. Source data are provided as a Source Data file.

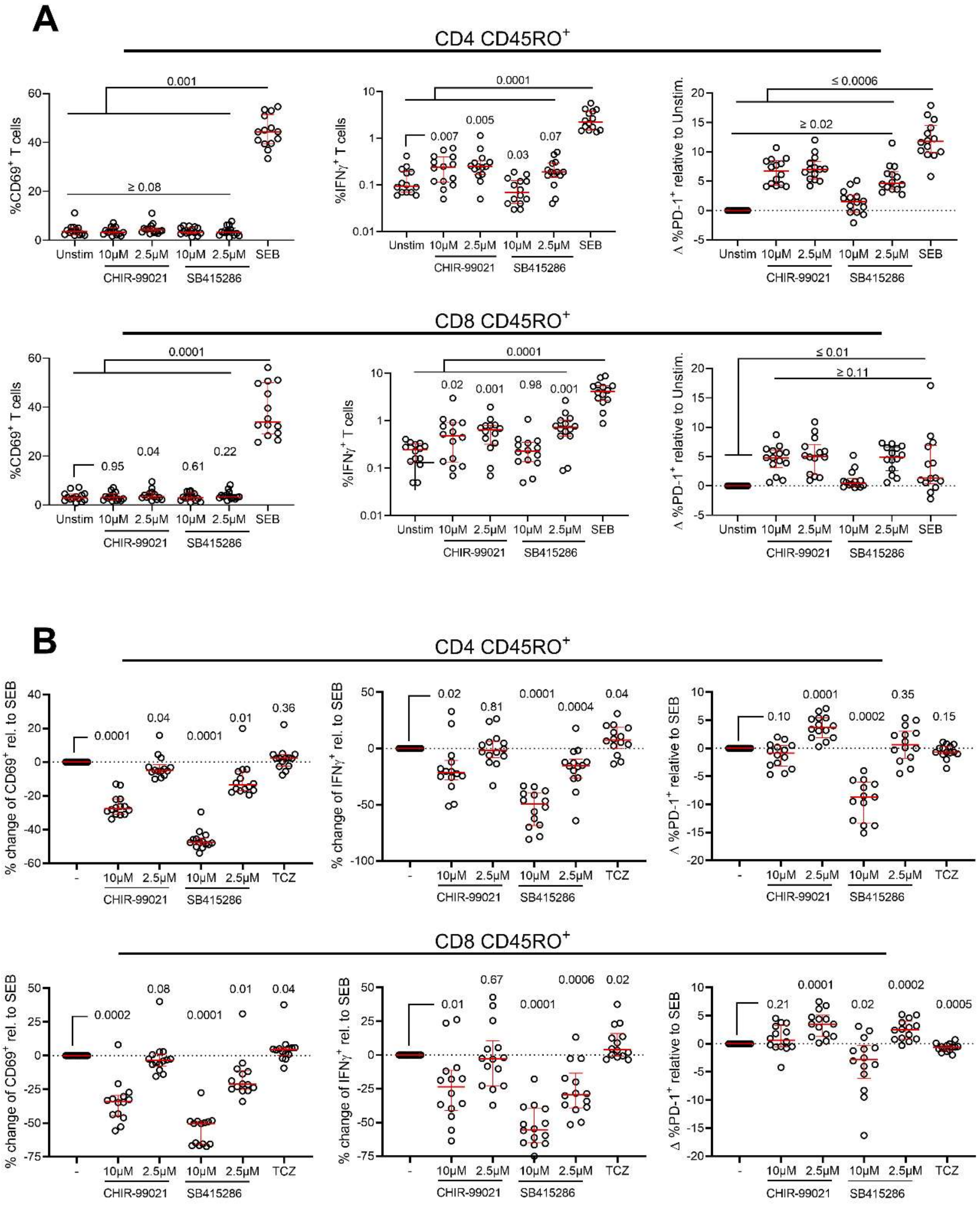

Supplementary Figure 11: T cell responses upon treatment with the GSK3 inhibitors CHIR-99021 and SB415286. (A) Percentages of CD69+, IFNγ+ and difference in percentages of PD1+ memory CD4 and CD8 T cells upon stimulation with CHIR-99021, SB415286 or SEB compared to unstimulated condition. (B) Difference in percentages of CD69+, IFNγ+ and PD1+ memory CD4 and CD8 T cells upon stimulation with SEB+ CHIR-99021, SEB+SB415286 or SEB+TCZ compared to SEB alone. Comparison between stimulation conditions in 14 ART-treated people living with HIV was performed by Wilcoxon paired test. Source data are provided as a Source Data file.

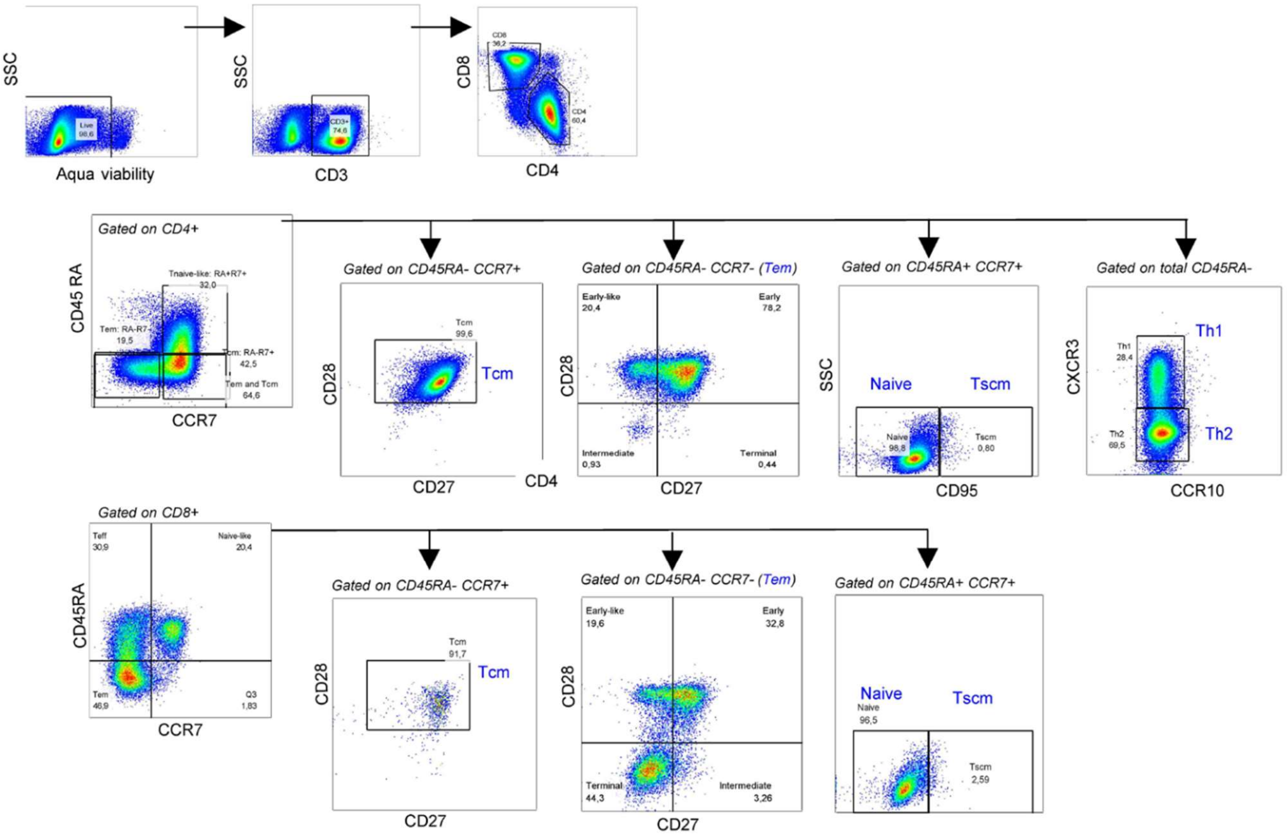

Supplementary Figure 12: Gating strategy of T cell subsets phenotyping.

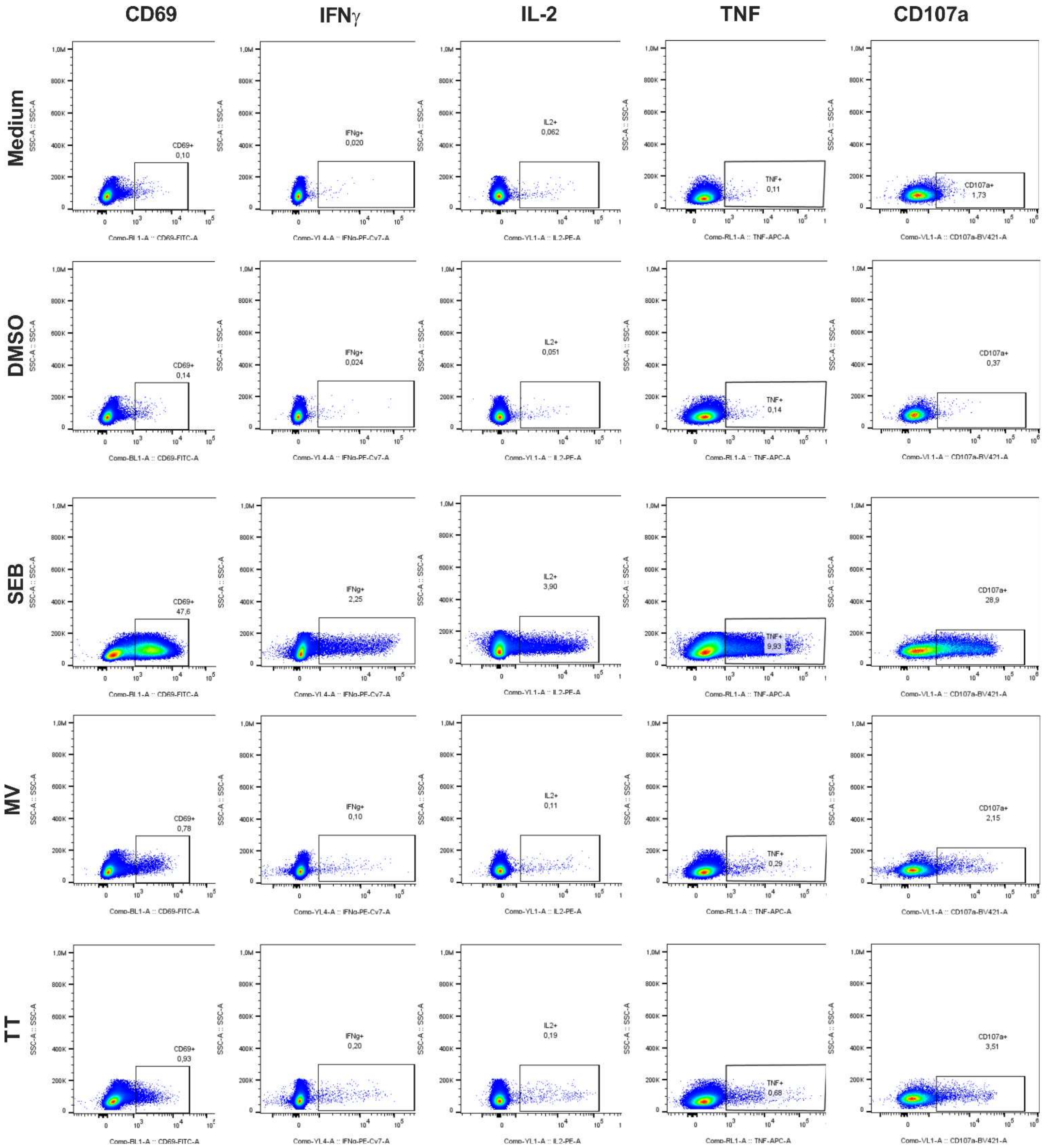

Supplementary Figure 13: CD69, IFNγ, IL-2, TNF and CD107a gating of intracellular cytokine staining experiment for stimuli and controls conditions.

Supplementary Table 1: Characteristics of study participants

	Uninfected	HIV-infected
N	34	33
Age in years -Median [range]	41 [23-64]	45 [23-60]
Gender ratio (M:F)	16:18	27:6
CMV seropositive	18	30
Time on ART in years – Median [IQR]	-	4.5 [3-13]
Pre-ART[a] plasma virus load (Log$_{10}$ HIV RNA copies) – Median [IQR]	-	4.79 [4.45-5.62]
Post-ART[b] plasma virus load (Log$_{10}$ HIV RNA copies)	-	<1.3
Pre-ART[a] CD4 T cell count (Cells/µl) – Median [IQR]	-	240 [67-392]
Post-ART[b] CD4 T cell count (Cells/µl) – Median [IQR]	-	748 [585 – 917]
Pre-ART[a] CD4:CD8 T cell ratio – Median [IQR]	-	0.40 [0.18-0.62]
Post-ART[b] CD4:CD8 T cell ratio – Median [IQR]	-	1.0 [0.8-1.35]

[a] Pre-ART measurements were performed on the day of ART start. [b] Post-ART measurements were performed at study entry. IQR: interquartile range.

Supplementary Table 2: Multivariate analysis of recall T cell responses with Benjamini-Hochberg correction for multiple testing, related to Figure 1

**Antigen**	**Variable**	***P***	***P*.adj**
MV	CD4 CD69+	0.003780	0.010710
MV	CD4 CD107+	0.303000	0.454500
MV	CD4 IFNγ+	0.000681	0.003157
MV	CD4 IL2+	0.012700	0.029441
MV	CD4 TNF+	0.004910	0.012623
MV	CD8 CD69+	0.002400	0.008160
MV	CD8 CD107+	0.885000	0.921122
MV	CD8 IFNγ+	0.004950	0.012623
MV	CD8 IL2+	0.164000	0.298714
MV	CD8 TNF+	0.001200	0.005100
MV	CD3 CD69+	0.000532	0.002713
MV	CD3 CD107+	0.455000	0.595000
MV	CD3 IFNγ+	0.000046	0.000469
MV	CD3 IL2+	0.000006	0.000162
MV	CD3 TNF+	0.000020	0.000251
MV	CD3+ Ki67+	0.000287	0.002091
MV	CD3- Ki67+	0.000336	0.002142
TT	CD4 CD69+	0.384000	0.529297
TT	CD4 CD107+	0.930000	0.948600
TT	CD4 IFNγ+	0.479000	0.610725
TT	CD4 IL2+	0.597000	0.724929
TT	CD4 TNF+	0.829000	0.906064
TT	CD8 CD69+	0.032100	0.068213
TT	CD8 CD107+	0.652000	0.762682
TT	CD8 IFNγ+	0.342000	0.484500
TT	CD8 IL2+	0.056200	0.110238
TT	CD8 TNF+	0.835000	0.906064
TT	CD3 CD69+	0.074700	0.141100
TT	CD3 CD107+	0.658000	0.762682
TT	CD3 IFNγ+	0.207000	0.329906
TT	CD3 IL2+	0.187000	0.307645
TT	CD3 TNF+	0.581000	0.722707
TT	CD3+ Ki67+	0.419000	0.562342
TT	CD3- Ki67+	0.171000	0.300724
SEB	CD4 CD69+	0.000073	0.000616
SEB	CD4 CD107+	0.328000	0.477943
SEB	CD4 IFNγ+	0.221000	0.341545
SEB	CD4 IL2+	0.037700	0.076908
SEB	CD4 TNF+	0.182000	0.307645
SEB	CD8 CD69+	0.000012	0.000202
SEB	CD8 CD107+	0.804000	0.906064
SEB	CD8 IFNγ+	0.001510	0.005924
SEB	CD8 IL2+	0.003720	0.010710
SEB	CD8 TNF+	1.000000	1.000000
SEB	CD3 CD69+	0.000004	0.000162
SEB	CD3 CD107+	0.868000	0.921122
SEB	CD3 IFNγ+	0.008560	0.020789
SEB	CD3 IL2+	0.000388	0.002199
SEB	CD3 TNF+	0.027400	0.060757
SEB	CD3+ Ki67+	0.003450	0.010710
SEB	CD3- Ki67+	0.002330	0.008160

Supplementary Table 3: Multivariate analysis of plasma and cellular markers of inflammation and immune activation with Benjamini-Hochberg correction for multiple testing, related to Figure 2

**Variable**	***P***	***P*.adj**
IFNγ	3.92E-10	8.62E-09
TNF	6.22E-08	6.84E-07
IL8	1.13E-06	8.29E-06
CD4 CD57+	7.12E-06	3.92E-05
IL-4	8.67E-05	0.000381
IFABP	0.000343	0.001254
IL-10	0.000430	0.001254
sCD14	0.000456	0.001254
IL-6	0.000609	0.001489
IL-12	0.002690	0.005918
CD4 HLA-DR+	0.010500	0.021000
CD4 PD1+	0.014600	0.026767
CXCL13	0.016000	0.027077
sCD163	0.037300	0.058614
CD8 HLA-DR+	0.042800	0.062773
CRP	0.085700	0.117838
CD8 CD57+	0.130000	0.168235
Mono	0.160000	0.195556
CD56hi	0.353000	0.408737
CD8 PD1+	0.596000	0.635905
LBP	0.607000	0.635905
MIP1B	0.660000	0.660000

Supplementary Table 4: Two-tailed comparisons of study variables between the total HIV-infected or uninfected group and respective subgroups for transcriptome analysis

		**Median of vaviables**				**Mann-Whitney *P***	
		**HU RNAseq**	**HIV RNAseq**	**All HU**	**All HIV**		
		**A**	**B**	**C**	**D**	***P* A vs C**	***P* B vs D**
**Plasma markers**	CRP	3410.0	4332.9	2196.5	3409.1	0.95550	0.97167
	CXCL13	42.4	41.6	33.8	42.0	0.83529	0.89912
	IFABP	785.4	1248.5	958.9	1375.5	0.58721	0.47401
	IFNγ	8.2	40.4	4.9	39.0	0.68804	0.93119
	IL4	0.3	0.6	0.4	0.6	0.77185	0.77185
	IL6	3.7	4.8	2.5	4.4	0.85905	0.69597
	IL8	3.1	5.7	3.1	5.0	0.74111	0.65827
	IL10	2.4	4.6	2.4	4.5	0.98785	0.90707
	IL12	4.2	5.0	3.2	6.4	0.62166	0.66549
	LBP	29940.7	22129.6	24833.6	23469.7	0.60836	0.86714
	MIP1B	10.5	13.5	10.5	13.0	0.57314	0.46760
	TNF	5.4	15.7	3.4	15.7	0.61403	0.86669
	sCD14	859.0	1197.1	985.7	1180.4	0.38487	0.45537
	sCD163	235.6	315.5	266.2	371.2	0.92328	0.60130
**Cell subsets freq.**	%CD4_DR+	2.7	3.5	2.8	3.8	0.64440	0.60130
	%CD4_PD1+	16.5	18.6	16.7	21.5	0.40760	0.38486
	%CD4_CD57+	0.8	4.6	0.4	3.4	0.45537	0.54591
	%CD8_DR+	3.8	5.5	4.4	5.5	0.76477	0.78030
	%CD8_PD1+	15.3	20.9	17.5	20.4	0.53246	0.54591
	%CD8_CD57+	4.5	7.8	4.3	6.3	0.40720	0.41947
	%CD56high	3.2	2.7	3.1	2.7	0.96361	0.94746
	%MONO	17.5	13.7	17.5	12.4	0.51263	0.91572
**MV in vitro resp.**	MV_CD4_CD69+	0.25	0.08	0.25	0.11	0.23638	0.52196
	MV_CD4_IFNg+	0.01	0.00	0.01	0.00	0.90501	0.60116
	MV_CD4_IL2+	0.02	0.01	0.02	0.01	0.97083	0.58583
	MV_CD4_TNF+	0.07	0.02	0.06	0.02	0.68324	0.98609
	MV_CD8_CD69+	0.03	0.01	0.06	0.01	0.45321	0.82902
	MV_CD8_CD107a+	0.12	0.09	0.19	0.23	0.18001	0.16825
	MV_CD8_IFNg+	0.01	0.00	0.01	0.00	0.97671	0.94753
	MV_CD8_IL2+	0.01	0.01	0.01	0.00	0.69992	0.39326
	MV_CD8_TNF+	0.02	0.00	0.02	0.00	0.84300	0.55931
	MV_CD3_CD69+	0.17	0.07	0.21	0.07	0.32032	0.65262
	MV_CD3_CD107a+	0.07	0.08	0.26	0.18	0.23062	0.23062
	MV_CD3_IFNg+	0.04	0.00	0.04	0.00	0.75936	0.90683
	MV_CD3_IL2+	0.07	0.02	0.07	0.01	0.82565	0.46939
	MV_CD3_TNF+	0.11	0.03	0.11	0.01	0.94557	0.71698
	MV_CD3+_Ki67+	0.23	0.10	0.19	0.07	0.87146	0.80610
	MV_CD3-_Ki67+	0.47	0.14	0.37	0.10	0.41688	0.53604
**TT in vitro resp.**	TT_CD4_CD69+	1.04	0.28	0.77	0.38	0.06352	0.10758
	TT_CD4_IFNg+	0.05	0.02	0.05	0.02	0.39222	0.33575
	TT_CD4_IL2+	0.16	0.07	0.10	0.08	0.15290	0.15290
	TT_CD4_TNF+	0.36	0.10	0.21	0.15	0.21361	0.26699
	TT_CD8_CD69+	0.98	0.07	0.39	0.09	0.08072	0.16455
	TT_CD8_CD107a+	0.93	0.18	0.37	0.35	0.06572	0.12948
	TT_CD8_IFNg+	0.07	0.01	0.03	0.02	0.17349	0.13408
	TT_CD8_IL2+	0.01	0.00	0.01	0.01	0.09555	0.23624
	TT_CD8_TNF+	0.16	0.02	0.10	0.06	0.58362	0.72275
	TT_CD3_CD69+	0.96	0.18	0.75	0.28	0.07419	0.12729
	TT_CD3_CD107a+	0.84	0.20	0.47	0.41	0.07336	0.09076
	TT_CD3_IFNg+	0.06	0.01	0.06	0.03	0.28249	0.23167
	TT_CD3_IL2+	0.16	0.03	0.09	0.05	0.13563	0.14219
	TT_CD3_TNF+	0.32	0.14	0.21	0.16	0.39206	0.41225
	TT_CD3+_Ki67+	0.18	0.11	0.13	0.11	0.77896	0.99523
	TT_CD3-_Ki67+	0.99	0.39	0.61	0.34	0.45510	0.79754

Supplementary Table 4 (continued)

		Median of vaviables				Mann-Whitney *P*	
		**HU RNAseq**	**HIV RNAseq**	**All HU**	**All HIV**		
		**A**	**B**	**C**	**D**	***P* A vs C**	***P* B vs D**
**SEB in vitro resp.**	SEB_CD4_CD69+	45.29	34.20	43.54	34.74	0.98573	0.94771
	SEB_CD4_IFNg+	1.85	2.16	1.55	1.85	0.72487	0.96671
	SEB_CD4_IL2+	5.02	4.90	4.69	4.01	0.72487	0.81608
	SEB_CD4_TNF+	9.70	9.05	9.55	8.33	0.98572	>0,999999
	SEB_CD8_CD69+	33.70	14.87	31.92	14.32	0.94770	0.90980
	SEB_CD8_CD107a+	24.98	22.63	22.13	23.58	0.77923	0.46243
	SEB_CD8_IFNg+	2.39	4.96	2.21	3.93	0.39922	0.53063
	SEB_CD8_IL2+	0.83	0.51	0.61	0.40	0.97619	0.63706
	SEB_CD8_TNF+	3.79	3.41	2.97	3.08	0.72487	0.63733
	SEB_CD3_CD69+	42.20	25.84	40.18	25.63	0.79755	0.85330
	SEB_CD3_CD107a+	21.72	16.13	20.13	17.67	0.79761	0.57063
	SEB_CD3_IFNg+	1.99	3.45	1.99	3.31	0.49970	0.63733
	SEB_CD3_IL2+	3.80	2.90	3.75	2.38	0.81608	0.90980
	SEB_CD3_TNF+	8.23	6.87	7.54	6.18	>0,999999	0.91923
	SEB_CD3+_Ki67+	8.18	5.57	7.98	4.57	0.53855	0.49216
	SEB_CD3-_Ki67+	8.66	3.59	8.38	3.03	0.63733	0.70704

Supplementary Table 5: Cell sorting yields

Study participant	stimulation condition	T cell subset	Cell number sorted	RNA concentration (ng/ul)
HIV01	Unstim	CD4+CD45RO+CD69-CD154-	100000	2.26
HIV01	MV	CD4+CD45RO+CD69-CD154-	220000	5.08
HIV01	TT	CD4+CD45RO+CD69-CD154-	190000	4.2
HIV01	MV	CD4+CD69+CD154+	5800	OOR
HIV01	TT	CD4+CD69+CD154+	3720	OOR
HIV02	Unstim	CD4+CD45RO+CD69-CD154-	130000	NA
HIV02	MV	CD4+CD45RO+CD69-CD154-	280000	4.19
HIV02	TT	CD4+CD45RO+CD69-CD154-	210000	3.81
HIV02	MV	CD4+CD69+CD154+	5700	OOR
HIV02	TT	CD4+CD69+CD154+	2300	OOR
HIV04	Unstim	CD4+CD45RO+CD69-CD154-	98000	1.71
HIV04	MV	CD4+CD45RO+CD69-CD154-	490000	8.24
HIV04	TT	CD4+CD45RO+CD69-CD154-	500000	IQ
HIV04	MV	CD4+CD69+CD154+	21000	OOR
HIV04	TT	CD4+CD69+CD154+	25200	OOR
HIV05	MV	CD4+CD69+CD154+	37640	0.8
HIV05	TT	CD4+CD69+CD154+	15500	OOR
HIV06	Unstim	CD4+CD45RO+CD69-CD154-	70000	1.13
HIV06	MV	CD4+CD45RO+CD69-CD154-	440000	6
HIV06	TT	CD4+CD45RO+CD69-CD154-	410000	5.92
HIV06	MV	CD4+CD69+CD154+	34000	OOR
HIV06	TT	CD4+CD69+CD154+	28000	OOR
HIV07	Unstim	CD4+CD45RO+CD69-CD154-	94000	2.03
HIV07	MV	CD4+CD45RO+CD69-CD154-	440000	8.2
HIV07	TT	CD4+CD45RO+CD69-CD154-	400000	7.2
HIV07	MV	CD4+CD69+CD154+	6870	OOR
HIV07	TT	CD4+CD69+CD154+	15640	OOR
HIV08	Unstim	CD4+CD45RO+CD69-CD154-	63700	1.33
HIV08	MV	CD4+CD45RO+CD69-CD154-	270000	3.61
HIV08	TT	CD4+CD45RO+CD69-CD154-	290000	3.86
HIV08	MV	CD4+CD69+CD154+	4120	OOR
HIV08	TT	CD4+CD69+CD154+	5670	OOR
HIV09	Unstim	CD4+CD45RO+CD69-CD154-	84000	1.25
HIV09	MV	CD4+CD45RO+CD69-CD154-	437000	NA
HIV09	TT	CD4+CD45RO+CD69-CD154-	374000	5.44
HIV09	MV	CD4+CD69+CD154+	3070	OOR
HIV09	TT	CD4+CD69+CD154+	1970	OOR
HIV10	Unstim	CD4+CD45RO+CD69-CD154-	240000	3.62
HIV10	MV	CD4+CD45RO+CD69-CD154-	400000	6.96
HIV10	TT	CD4+CD45RO+CD69-CD154-	380000	5.36
HIV10	MV	CD4+CD69+CD154+	5120	OOR
HIV10	TT	CD4+CD69+CD154+	4880	OOR
HIV100	Unstim	CD4+CD45RO+CD69-CD154-	220000	5.04
HIV100	MV	CD4+CD45RO+CD69-CD154-	550000	10.6
HIV100	TT	CD4+CD45RO+CD69-CD154-	470000	8.84
HIV100	MV	CD4+CD69+CD154+	8300	OOR
HIV100	TT	CD4+CD69+CD154+	3500	OOR
HIV11	Unstim	CD4+CD45RO+CD69-CD154-	180000	NA
HIV11	MV	CD4+CD45RO+CD69-CD154-	357000	7.48
HIV11	TT	CD4+CD45RO+CD69-CD154-	400000	8.24
HIV11	MV	CD4+CD69+CD154+	63000	OOR
HIV11	TT	CD4+CD69+CD154+	51000	0.8
HIV12	Unstim	CD4+CD45RO+CD69-CD154-	185000	0.8
HIV12	MV	CD4+CD45RO+CD69-CD154-	370000	4.12
HIV12	TT	CD4+CD45RO+CD69-CD154-	396000	4.12
HIV12	MV	CD4+CD69+CD154+	20300	OOR
HIV12	TT	CD4+CD69+CD154+	15500	OOR
HIV18	Unstim	CD4+CD45RO+CD69-CD154-	100000	NA
HIV18	MV	CD4+CD45RO+CD69-CD154-	420000	8.52
HIV18	TT	CD4+CD45RO+CD69-CD154-	400000	8.04
HIV18	MV	CD4+CD69+CD154+	8000	OOR
HIV18	TT	CD4+CD69+CD154+	7490	OOR
HIV19	Unstim	CD4+CD45RO+CD69-CD154-	290000	3.2
HIV19	MV	CD4+CD45RO+CD69-CD154-	670000	7.28
HIV19	TT	CD4+CD45RO+CD69-CD154-	460000	6.12
HIV19	MV	CD4+CD69+CD154+	30000	OOR
HIV19	TT	CD4+CD69+CD154+	20000	OOR

Supplementary Table 5 (continued)

Study participant	stimulation condition	T cell subset	Cell number sorted	RNA concentration (ng/ul)
HIV20	MV	CD4+CD45RO+CD69-CD154-	300000	5.2
HIV20	TT	CD4+CD45RO+CD69-CD154-	350000	4.36
HIV20	MV	CD4+CD69+CD154+	3980	OOR
HIV20	TT	CD4+CD69+CD154+	5000	OOR
HIV23	Unstim	CD4+CD45RO+CD69-CD154-	120000	2.12
HIV23	MV	CD4+CD45RO+CD69-CD154-	580000	10.9
HIV23	TT	CD4+CD45RO+CD69-CD154-	635000	11.8
HIV23	MV	CD4+CD69+CD154+	23400	OOR
HIV23	TT	CD4+CD69+CD154+	20890	OOR
HIV24	Unstim	CD4+CD45RO+CD69-CD154-	160000	2
HIV24	MV	CD4+CD45RO+CD69-CD154-	420000	NA
HIV24	TT	CD4+CD45RO+CD69-CD154-	280000	7.56
HIV24	MV	CD4+CD69+CD154+	6600	OOR
HIV24	TT	CD4+CD69+CD154+	4200	OOR
HU02	Unstim	CD4+CD45RO+CD69-CD154-	264000	6.16
HU02	MV	CD4+CD45RO+CD69-CD154-	575000	5.36
HU02	TT	CD4+CD45RO+CD69-CD154-	470000	10.2
HU02	MV	CD4+CD69+CD154+	78000	1.08
HU02	TT	CD4+CD69+CD154+	90000	1.4
HU03	Unstim	CD4+CD45RO+CD69-CD154-	170000	2.98
HU03	MV	CD4+CD45RO+CD69-CD154-	280000	5.96
HU03	TT	CD4+CD45RO+CD69-CD154-	260000	4.2
HU03	MV	CD4+CD69+CD154+	32000	OOR
HU03	TT	CD4+CD69+CD154+	38000	OOR
HU04	Unstim	CD4+CD45RO+CD69-CD154-	98000	1.9
HU04	MV	CD4+CD45RO+CD69-CD154-	330000	5.8
HU04	TT	CD4+CD45RO+CD69-CD154-	270000	5.44
HU04	MV	CD4+CD69+CD154+	14000	OOR
HU04	TT	CD4+CD69+CD154+	8000	OOR
HU05	Unstim	CD4+CD45RO+CD69-CD154-	170000	3.38
HU05	MV	CD4+CD45RO+CD69-CD154-	840000	16.1
HU05	TT	CD4+CD45RO+CD69-CD154-	770000	12.2
HU06	Unstim	CD4+CD45RO+CD69-CD154-	100000	1.9
HU06	MV	CD4+CD45RO+CD69-CD154-	490000	NA
HU06	TT	CD4+CD45RO+CD69-CD154-	540000	9.76
HU06	MV	CD4+CD69+CD154+	29620	OOR
HU06	TT	CD4+CD69+CD154+	11620	OOR
HU09	Unstim	CD4+CD45RO+CD69-CD154-	340000	NA
HU09	MV	CD4+CD45RO+CD69-CD154-	470000	6.28
HU09	TT	CD4+CD45RO+CD69-CD154-	410000	6.52
HU09	MV	CD4+CD69+CD154+	18000	OOR
HU09	TT	CD4+CD69+CD154+	11000	OOR
HU10	Unstim	CD4+CD45RO+CD69-CD154-	49000	1.07
HU10	MV	CD4+CD45RO+CD69-CD154-	245000	5.28
HU10	TT	CD4+CD45RO+CD69-CD154-	280000	6.24
HU10	MV	CD4+CD69+CD154+	38600	1.06
HU10	TT	CD4+CD69+CD154+	19820	OOR
HU12	Unstim	CD4+CD45RO+CD69-CD154-	100000	2.37
HU12	MV	CD4+CD45RO+CD69-CD154-	620000	12.6
HU12	TT	CD4+CD45RO+CD69-CD154-	710000	NA
HU12	MV	CD4+CD69+CD154+	11610	OOR
HU12	TT	CD4+CD69+CD154+	16860	OOR
HU13	Unstim	CD4+CD45RO+CD69-CD154-	61580	1.02
HU13	MV	CD4+CD45RO+CD69-CD154-	268000	5.12
HU13	TT	CD4+CD45RO+CD69-CD154-	247000	4.08
HU13	MV	CD4+CD69+CD154+	11290	OOR
HU13	TT	CD4+CD69+CD154+	5640	OOR
HU15	Unstim	CD4+CD45RO+CD69-CD154-	134000	2.29
HU15	MV	CD4+CD45RO+CD69-CD154-	670000	7.52
HU15	TT	CD4+CD45RO+CD69-CD154-	698000	9.6
HU15	MV	CD4+CD69+CD154+	3460	OOR
HU15	TT	CD4+CD69+CD154+	3100	OOR
HU17	Unstim	CD4+CD45RO+CD69-CD154-	296000	6.32
HU17	MV	CD4+CD45RO+CD69-CD154-	730000	14.1
HU17	TT	CD4+CD45RO+CD69-CD154-	700000	13
HU17	MV	CD4+CD69+CD154+	10430	OOR
HU17	TT	CD4+CD69+CD154+	9440	OOR

Supplementary Table 5 (continued)

Study participant	stimulation condition	T cell subset	Cell number sorted	RNA concentration (ng/ul)
HU18	Unstim	CD4+CD45RO+CD69-CD154-	240000	8.12
HU18	MV	CD4+CD45RO+CD69-CD154-	438000	NA
HU18	TT	CD4+CD45RO+CD69-CD154-	NA	8.36
HU18	MV	CD4+CD69+CD154+	54000	0.8
HU18	TT	CD4+CD69+CD154+	NA	OOR
HU19	Unstim	CD4+CD45RO+CD69-CD154-	249000	3.73
HU19	MV	CD4+CD45RO+CD69-CD154-	600000	10.2
HU19	TT	CD4+CD45RO+CD69-CD154-	370000	NA
HU19	MV	CD4+CD69+CD154+	137000	2.94
HU19	TT	CD4+CD69+CD154+	51800	1
HU20	Unstim	CD4+CD45RO+CD69-CD154-	340000	8.32
HU20	MV	CD4+CD45RO+CD69-CD154-	720000	14.3
HU20	TT	CD4+CD45RO+CD69-CD154-	750000	16.7
HU20	MV	CD4+CD69+CD154+	46000	0.92
HU20	TT	CD4+CD69+CD154+	44000	0.84
HU21	Unstim	CD4+CD45RO+CD69-CD154-	450000	6.2
HU21	MV	CD4+CD45RO+CD69-CD154-	900000	11.8
HU21	TT	CD4+CD45RO+CD69-CD154-	1000000	18.2
HU21	MV	CD4+CD69+CD154+	65000	1.06
HU21	TT	CD4+CD69+CD154+	60000	1.25
HU22	Unstim	CD4+CD45RO+CD69-CD154-	346000	5.44
HU22	MV	CD4+CD45RO+CD69-CD154-	660000	NA
HU22	TT	CD4+CD45RO+CD69-CD154-	738000	10.8
HU22	MV	CD4+CD69+CD154+	27240	OOR
HU22	TT	CD4+CD69+CD154+	15690	OOR
HU23	Unstim	CD4+CD45RO+CD69-CD154-	47800	NA
HU23	MV	CD4+CD45RO+CD69-CD154-	250000	4.72
HU23	TT	CD4+CD45RO+CD69-CD154-	256000	3.3
HU23	MV	CD4+CD69+CD154+	8870	OOR
HU23	TT	CD4+CD69+CD154+	8000	OOR
HU24	Unstim	CD4+CD45RO+CD69-CD154-	51000	NA
HU24	MV	CD4+CD45RO+CD69-CD154-	320000	4.96
HU24	TT	CD4+CD45RO+CD69-CD154-	270000	4.84
HU24	MV	CD4+CD69+CD154+	19500	OOR
HU24	TT	CD4+CD69+CD154+	12000	OOR
HU25	Unstim	CD4+CD45RO+CD69-CD154-	100000	2
HU25	MV	CD4+CD45RO+CD69-CD154-	290000	6.04
HU25	TT	CD4+CD45RO+CD69-CD154-	350000	7.96
HU25	MV	CD4+CD69+CD154+	51000	1
HU25	TT	CD4+CD69+CD154+	42000	0.8
