# Supplementary material for: Chronic inflammation degrades CD4 T cell immunity to prior vaccines in treated HIV infection
Source: Nat Commun. 2024 Nov 25;15:10200. doi: 10.1038/s41467-024-54605-3 (PMC11589758; doi:10.1038/s41467-024-54605-3)
Supplement: Supplementary file 2 — Description of Additional Supplementary Files [file 41467_2024_54605_MOESM2_ESM.pdf]

Description of Additional Supplementary Files

File Name: Supplementary Data 1
Description: DEGs unique to either HIV or HU groups, related to Figure 5.

File Name: Supplementary Data 2
Description: Spreadsheet of gene-level overrepresentation analysis for all sorted T cell subsets after MV or TT stimulation, related to Figure 5.

File Name: Supplementary Data 3
Description: Enrichment scores and adjusted P values of all MSigDB VAX gene sets significantly enriched in either HIV-infected or uninfected groups, related to Figure 6.

File Name: Supplementary Data 4
Description: Enrichment scores and adjusted P values of all MSigDB Hallmark gene sets significantly enriched in either HIV-infected or uninfected groups, related to Figure 6.
