# Supplementary material for: Chronic inflammation degrades CD4 T cell immunity to prior vaccines in treated HIV infection
Source: Nat Commun. 2024 Nov 25;15:10200. doi: 10.1038/s41467-024-54605-3 (PMC11589758; doi:10.1038/s41467-024-54605-3)
Supplement: Supplementary file 7 — Reporting Summary [file 41467_2024_54605_MOESM7_ESM.pdf]

nature portfolio

Corresponding author(s): Krystelle Nganou-Makamdop
Last updated by author(s): 16 Oct 2024

Reporting Summary

Nature Portfolio wishes to improve the reproducibility of the work that we publish. This form provides structure for consistency and transparency in reporting. For further information on Nature Portfolio policies, see our Editorial Policies and the Editorial Policy Checklist.

Statistics

For all statistical analyses, confirm that the following items are present in the figure legend, table legend, main text, or Methods section.

n/a	Confirmed	
☐	☒	The exact sample size (*n*) for each experimental group/condition, given as a discrete number and unit of measurement
☐	☒	A statement on whether measurements were taken from distinct samples or whether the same sample was measured repeatedly
☐	☒	The statistical test(s) used AND whether they are one- or two-sided<br>*Only common tests should be described solely by name; describe more complex techniques in the Methods section.*
☐	☒	A description of all covariates tested
☐	☒	A description of any assumptions or corrections, such as tests of normality and adjustment for multiple comparisons
☒	☐	A full description of the statistical parameters including central tendency (e.g. means) or other basic estimates (e.g. regression coefficient) AND variation (e.g. standard deviation) or associated estimates of uncertainty (e.g. confidence intervals)
☒	☐	For null hypothesis testing, the test statistic (e.g. *F*, *t*, *r*) with confidence intervals, effect sizes, degrees of freedom and *P* value noted<br>*Give P values as exact values whenever suitable.*
☒	☐	For Bayesian analysis, information on the choice of priors and Markov chain Monte Carlo settings
☒	☐	For hierarchical and complex designs, identification of the appropriate level for tests and full reporting of outcomes
☐	☒	Estimates of effect sizes (e.g. Cohen's *d*, Pearson's *r*), indicating how they were calculated

Our web collection on statistics for biologists contains articles on many of the points above.

Software and code

Policy information about availability of computer code

Data collection	Flow cytometry analysis of cells was performed on an ATTuneNeXT instrument with associated software .<br>qPCR data were generated on a 7500 real time PCR system (Applied Biosystems)<br>Luminex data were acquired on a MagPix aquisition software<br>Sequencing data were generated on a NovaSeq 6000 (Illumina)
Data analysis	Flow cytometry data were analyzed with a FlowJov10 software and Graphpad PRISM.<br>qPCR data were analyzed with a 7500v2.3 software<br>Correlations between the various assay measures was perform by Spearman correlation analysis in R and plotted using the R Studio packages "corrplot" and "RColorBrewer"<br>Analysis of sequencing data was done with STAR aligner (version 2.5.3a), HTSeq (version 0.9.1), DESeq2 (in R)<br>Codes for SEARCHLIGHT analysis of RNAseq data are available on https://github.com/Searchlight2/Searchlight2.

For manuscripts utilizing custom algorithms or software that are central to the research but not yet described in published literature, software must be made available to editors and reviewers. We strongly encourage code deposition in a community repository (e.g. GitHub). See the Nature Portfolio guidelines for submitting code & software for further information.

Data

Policy information about availability of data

All manuscripts must include a data availability statement. This statement should provide the following information, where applicable:

- Accession codes, unique identifiers, or web links for publicly available datasets
- A description of any restrictions on data availability
- For clinical datasets or third party data, please ensure that the statement adheres to our policy

This study did not generate new unique reagents. Whole transcriptome sequencing data generated in this study are available at Gene Expression Omnibus (GEO) under the series accession number GSE273967. Source data are provided with this paper.

Research involving human participants, their data, or biological material

Policy information about studies with human participants or human data. See also policy information about sex, gender (identity/presentation), and sexual orientation and race, ethnicity and racism.

Reporting on sex and gender	Our study included both male and female participants, with sex assigned based on self-report. and no sex-dimorphic effects are reported. While we aimed for a balanced sex distribution among the recruited sex participants, the study was not designed to assess sex-related effects and the participants consent did not cover reporting of sex-related data.
Reporting on race, ethnicity, or other socially relevant groupings	All participans were of central european origin i.e. caucasian ethnicity. The median age was 42 years, ranging between 23 to 64 years. 43 were male and 24 were female.
Population characteristics	33 participants had been previously diagnosed with HIV infection and were on antiretroviral therapy at the time of study.
Recruitment	Participants with HIV infection were recruited through the HIV outpatient clinic of the Universitätsklinikum Erlagen. Possibility of participation to the study was presented by the handling physician upon routine visit and, upon interest in partication in the study, blood samples were collected by the nurse staff after provision of a signed consent form. For the control group, participants were approached by means of a flyer at the department of transfusion medicine and persons who were interested in participating in the study could contact the leading study investigator for scheduled visit.
Ethics oversight	The study protocol was approved by the ethics committees of the Universitätsklinikum Erlangen (235_18B) and carried out in compliance with institutional guidelines. All participants gave written, informed consent in accordance with the Declaration of Helsinki.

Note that full information on the approval of the study protocol must also be provided in the manuscript.

Field-specific reporting

Please select the one below that is the best fit for your research. If you are not sure, read the appropriate sections before making your selection.

☒ Life sciences ☐ Behavioural & social sciences ☐ Ecological, evolutionary & environmental sciences

For a reference copy of the document with all sections, see nature.com/documents/nr-reporting-summary-flat.pdf

Life sciences study design

All studies must disclose on these points even when the disclosure is negative.

Sample size	Sample size was not pre-determined
Data exclusions	No data were excluded
Replication	Aside from the sequencing that was perform in a single run, all other assays were performed over various experiments and comparisons in outcome of the various experiments of a given assay did not show different profiles.
Randomization	Samples were randomly pulled out for testing and caring for that participants from both groups were represented in any given experiment
Blinding	Investigators were not blinded in order to make it possible to include samples from participants of both groups in any assessment.

Reporting for specific materials, systems and methods

We require information from authors about some types of materials, experimental systems and methods used in many studies. Here, indicate whether each material, system or method listed is relevant to your study. If you are not sure if a list item applies to your research, read the appropriate section before selecting a response.

Materials & experimental systems

n/a	Involved in the study
☐	☒ Antibodies
☒	☐ Eukaryotic cell lines
☒	☐ Palaeontology and archaeology
☒	☐ Animals and other organisms
☒	☐ Clinical data
☒	☐ Dual use research of concern
☒	☐ Plants

Methods

n/a	Involved in the study
☐	☐ ChIP-seq
☐	☒ Flow cytometry
☐	☐ MRI-based neuroimaging

Antibodies

Antibodies used

CD3-Cy7APC (BD, clone SP34-2); HLADR-TRPE (Invitrogen, clone TU36); CD8-BV570 (Biolegend, clone RPA-T8), CD4-CY55PE (Invitrogen, clone S3.5), PD1-BV421 (Biolegend, clone EH12.2H7), CD14-PE (BD, clone M5E2), CD19-AF700 (Biolegend, clone HIB19), CD56-FITC (BD, clone NCAM16.2), CD57-BV605 (clone QA17A04), CD3-Cy7APC (BD, clone SP34-2); CD8 Pacific Blue (BD, clone RPA-T8), CXCR3-BV421 (Biolegend, clone G025H7), CD27-BV711 (Biolegend, clone O323), CD57-FITC (Biolegend, clone HNK-1), CD8-PerCPCy55 (Biolegend, clone RPA-T8), CCR10-PE (Biolegend, clone 6588-5), CD4-PEAF700 (Invitrogen, clone S3.5), CCR7-PECy7 (Biolegend, clone G043H7), CD28-APC (Biolegend, clone 28.2), CD3-AF700 (Biolegend, clone UCHT1), CD45RA-APCCy7 (Biolegend, clone HI100),CD28 (BD, clone L293), anti-CD49d (BD, clone L25), CD107a-BV421 (Biolegend, clone H4A3), CD69-FITC (Biolegend, clone FN50), IFN-Cy7PE (Biolegend, clone B27), IL-2-PE (Biolegend, clone MQ1-17H2) and TNF-APC (BD, clone Mab11), CD3-PE (BD, clone SK7), Ki67-AF647 (BD, clone B56); CD154-PE (BD, clone TRAP), CD4-APCF750 (Biolegend, clone SK3), CD8-BV421 (Biolegend, clone RPA-T8), CD45RO-BV650 (Biolegend, clone UCHL1) and CD69-FITC (Biolegend, clone FN50). CD3-AF700 (Biolegend, clone UCHT1), CD4-FITC (Biolegend, clone RPA-T4), CD8-APC (Biolegend, clone RPA-T8), PD1-BV421 (Biolegend, clone EH12.2H7) and CD45RO-BV650 (Biolegend, clone UCHL-1), CD69-BV510 (Biolegend, clone FN50), IFNg-Cy7PE (Biolegend, clone B27), CD69-Cy7PE (Biolegend, clone FN50), IFNg-APC (Biolegend, clone 4S.B3).

Validation

Antibodies were validated by the supplier

Plants

Seed stocks

Not applicable

Novel plant genotypes

Not applicable

Authentication

Not applicable

ChIP-seq

Data deposition

☐ Confirm that both raw and final processed data have been deposited in a public database such as GEO.

☐ Confirm that you have deposited or provided access to graph files (e.g. BED files) for the called peaks.

Data access links
May remain private before publication.

Not applicable

Files in database submission

Not applicable

Genome browser session
(e.g. UCSC)

Not applicable

Methodology

Replicates

Not applicable

Sequencing depth

Not applicable

Antibodies	Not applicable
Peak calling parameters	Not applicable
Data quality	Not applicable
Software	Not applicable

Flow Cytometry

Plots

Confirm that:

- [x] The axis labels state the marker and fluorochrome used (e.g. CD4-FITC).
- [x] The axis scales are clearly visible. Include numbers along axes only for bottom left plot of group (a 'group' is an analysis of identical markers).
- [x] All plots are contour plots with outliers or pseudocolor plots.
- [x] A numerical value for number of cells or percentage (with statistics) is provided.

Methodology

Sample preparation	While blood cells were isolated from whole blood by density centrifugation and were preserved in liquid nitrogen until measurements
Instrument	AttuneNext (ThermoFisher)
Software	AttuneNext softwarre
Cell population abundance	Purity of post-sort fractions was determined by running isolated fractions again during test sorts
Gating strategy	Gating strategies included FSC-A vs SSC-A for lymphoctes; FSC-A vs FSC-H to exclude doublets and then live dead gate.

- [x] Tick this box to confirm that a figure exemplifying the gating strategy is provided in the Supplementary Information.

Magnetic resonance imaging

Experimental design

Design type	Not applicable
Design specifications	Not applicable
Behavioral performance measures	Not applicable

Acquisition

Imaging type(s)	Not applicable
Field strength	Not applicable
Sequence & imaging parameters	Not applicable
Area of acquisition	Not applicable

Diffusion MRI ☐ Used ☐ Not used

Preprocessing

Preprocessing software	Not applicable
Normalization	Not applicable
Normalization template	Not applicable
Noise and artifact removal	Not applicable
Volume censoring	Not applicable

Statistical modeling & inference

Model type and settings: Not applicable

Effect(s) tested: Not applicable

Specify type of analysis: ☐ Whole brain ☐ ROI-based ☐ Both

Statistic type for inference: Not applicable

(See Eklund et al. 2016)

Correction: Not applicable

Models & analysis

n/a	Involved in the study
☒	☐ Functional and/or effective connectivity
☒	☐ Graph analysis
☒	☐ Multivariate modeling or predictive analysis
